# Blood pressure response to standing is a strong determinant of masked hypertension in young to middle-age individuals

**DOI:** 10.1097/HJH.0000000000003188

**Published:** 2022-08-23

**Authors:** Paolo Palatini, Lucio Mos, Marcello Rattazzi, Paolo Spinella, Andrea Ermolao, Olga Vriz, Francesca Battista, Francesca Saladini

**Affiliations:** a Department of Medicine - University of Padova, Padova; b San Antonio Hospital, San Daniele del Friuli; c Cittadella Town Hospital, Cittadella, Italy

**Keywords:** orthostatic, standing, reactivity, masked hypertension, ambulatory, epinephrine, sympathetic

## Abstract

**Objective:**

The pathophysiologic mechanisms of masked hypertension are still debated. The aim of this study was to investigate whether the blood pressure response to standing is a determinant of masked hypertension in young individuals.

**Design and methods:**

We studied 1078 individuals (mean age 33.2 ± 8.5 years) with stage-1 untreated hypertension at baseline. Orthostatic response was defined as the difference between six SBP measurements in the orthostatic and supine postures. People with a response more than 6.5 mmHg (upper decile) were defined as hyperreactors. After 3 months of follow-up, 24-h ambulatory BP was measured and the participants were classified as normotensives (*N* = 120), white-coat hypertensive individuals (*N* = 168), masked hypertensive individuals (*N* = 166) and sustained hypertensive individuals (*N* = 624). In 591 participants, 24-h urinary epinephrine was also measured.

**Results:**

Orthostatic response was an independent predictor of masked hypertension after 3 months (*P* = 0.001). In the whole group, the odds ratio for the Hyperreactors was 2.5 [95% confidence interval (95% CI) 1.5–4.0, *P* < 0.001]. In the participants stratified by orthostatic response and urinary epinephrine, the odds ratio for masked hypertension was 4.2 (95% CI, 1.8–9.9, *P* = 0.001) in the hyperreactors with epinephrine above the median and was 2.6 (95% CI, 0.9–7.3, *P* = 0.069) in those with epinephrine below the median. The association between orthostatic response and masked hypertension was confirmed in the cross-sectional analysis after 3 months (*P* < 0.001).

**Conclusion:**

The present findings indicate that hyperreactivity to standing is a significant determinant of masked hypertension. The odds ratio for masked hypertension was even quadrupled in people with an orthostatic response more than 6.5 mmHg and high urinary epinephrine suggesting a role of sympathoadrenergic activity in the pathogenesis of masked hypertension.

## INTRODUCTION

Masked hypertension is a condition characterized by normal office blood pressure (BP) and high BP outside the office, which can be identified with 24-h ambulatory BP monitoring (ABPM) or home BP monitoring. Masked hypertension has been suggested to be associated with hypertension-mediated organ damage such as left ventricular hypertrophy and carotid artery atherosclerosis [1,2]. Further, the IDACO (International Database of Ambulatory blood pressure in relation to Cardiovascular Outcome in a general population) study of 9691 individuals [3] and other studies [4,5] reported that masked hypertension might be an independent risk factor for the incidence of cardiovascular diseases irrespective of antihypertensive treatment status.

Because masked hypertension is a strong risk factor for adverse cardiovascular outcomes, clinicians should try to identify individuals who are at a high risk for this condition, with the goal of preventing cardiovascular events in this population. Clinical characteristics associated with masked hypertension have been found to be male sex, middle age, overweight or obesity, diabetes, smoking, regular alcohol drinking, high daytime physical activity [6–8] and an exaggerated BP response (EBPR) to exercise testing [9].

An alternative easier screening test would be clinically useful to differentiate between people with and without masked hypertension. Previous studies have suggested a possible association between orthostatic hypertension and masked hypertension [10,11]. In a cross-sectional study in a general population, Tabara *et al.*[11] found a significant relationship between orthostatic BP change and office-ambulatory daytime BP difference. In this population, the frequency of masked hypertension was significantly greater in individuals who showed orthostatic hypertension 3 min after standing [11]. These data suggest that measuring the BP response to standing might facilitate the identification of people with masked hypertension. The mechanisms underlying the link between orthostatic hypertension and masked hypertension are poorly understood. One possible mechanism may be enhanced sympathetic nervous system activity, a condition that has been found in both clinical entities [12,13].

Thus, the purpose of our study was to investigate whether the BP response to standing was a determinant of masked hypertension in a cohort of young-to-middle-age individuals screened for stage 1 hypertension participating in the HARVEST (hypertension and ambulatory recording Venetia study). To this end, the postural BP changes measured 3 months before and at the same time of the ambulatory BP assessment were used. Another purpose of this investigation was to establish whether this putative association was characterized by increased sympatho-adrenergic activity.

## MATERIALS AND METHODS

Study participants were 1078 participants from the HARVEST study, a prospective multicentre observational study, involving 17 centres in North East Italy [14,15]. According to the study criteria, patients enrolled were young to middle age, were screened for stage 1 hypertension (SBP 140–159 mmHg and/or DBP 90–99 mmHg), and had never been treated for the disease before enrolment. Patients with high cardiovascular risk, diabetes mellitus, previous cardiovascular events, renal impairment and secondary forms of hypertension were excluded. More details regarding recruitment criteria were previously published [14,15]. The present analysis was conducted in the participants who performed ABPM after 3 months of follow-up in the absence of antihypertensive treatment. The study was approved by the HARVEST Ethics Committee and by the Ethics Committee of the University of Padova and has been performed in accordance with the ethical standards as laid down in the 1964 Declaration of Helsinki and its later amendments. A written informed consent was given by all study participants.

### Procedures

According to the HARVEST study protocol [14,15], at the baseline, patients underwent physical examination, anthropometry, blood chemistry after an overnight fast to measure lipids and glucose, and urine collection. Data regarding medical history, family history of cardiovascular disease and lifestyle habits, including involvement in physical activity, smoking habits, coffee and alcohol consumption, were collected by means of a self-reported questionnaire [16,17]. At the initial evaluation, all individuals received general information about nonpharmacological measures by the HARVEST investigators, following the suggestions of current guidelines on the management of hypertensive patients.

#### Office blood pressure measurement

At entry, brachial office BP was measured with a mercury sphygmomanometer and a cuff of appropriate size, during two visits performed 2 weeks apart. At each visit, three supine measurements were taken after the participant had lain on the examination bed for a minimum of 5 min. After the supine data were collected, the participant assumed the upright position and three additional BP measurements were taken at 1-min intervals. The difference between the three orthostatic and the three supine measurements was calculated. The orthostatic BP response to standing at baseline was defined as the average of the two standing-lying BP differences obtained during the two baseline visits.

#### Ambulatory blood pressure monitoring

Ambulatory BP recording was performed at baseline using the A&D TM2420 model 7 (A&D, Tokyo, Japan) or ICR Spacelabs 90207 monitor (Spacelabs, Redmond, Washington, USA) devices. Both devices were previously validated and were shown to provide comparable results [15]. According to the HARVEST study protocol, measurements were taken every 10 min during the day (0600–2300 h) and every 15–30 min during the night (2300–0600 h). Participants were instructed to go to bed and to wake up according to our scheduled times. Patient's adherence was checked from the diary card. After 3 months, ABPM was repeated following the same procedures used at baseline.

At the baseline, urine was collected for epinephrine and norepinephrine measurement in 591 participants. Immediately after completion, volumes were measured and urine specimens were frozen (−20 °C) and then sent to the Coordinating Center in Padua. Here, epinephrine and norepinephrine were assessed by a HPLC method and normalized by 24-h creatinine output measured with the Jaffe method. All samples from a given participant were analysed in the same batch in duplicate.

#### Patients’ classification

Participants were categorized into four groups according to their supine office and average 24-h BP measured after 3 months. The cutoff values used to define normal and high BPs were 140/90 mmHg for office BP, 130/80 mmHg for 24-h BP and 135/85 mmHg for daytime BP. Using these cut-offs, we identified four different groups: People with normal office and 24-h BP (Normotensive individuals); People with high office BP and normal 24-h BP (White-coat hypertensive individuals); People with normal office BP and high 24-h BP (Masked hypertensive individuals); and People with high office and 24-h BP (Sustained hypertensive individuals). In addition, in the two-way analysis of covariance (ANCOVA) and logistic regression analyses, a binary classification was used by dividing the patients according to whether they had masked hypertension (Group 1) or not (Group 0). As in some studies, night-time (or sleep) BP has also been used as a criterion for masked hypertension given the independent predictive value of night-time BP for cardiovascular events [18]; in the present study, masked hypertension was also defined as isolated masked asleep hypertension (SBP ≥120 mmHg) or as a combination of elevated night-time SBP and/or elevated mean 24-h SBP [19].

#### Statistical analyses

In agreement with our previous report [20], an exaggerated BP reaction to standing was considered as the lower limit of the upper decile of the postural SBP change (> 6.5 mmHg). To study the possible relationship of orthostatic hypotension with masked hypertension, also, the decile at the lowest extreme of the postural change distribution (≤ -12.0 mmHg, hyporeactors, *N* = 109) was taken into account. Also, analyses on postural changes in DBP were performed. However, orthostatic DBP changes showed little or no association with masked hypertension, and thus, only results for SBP are presented here. Quantitative variables were reported as mean and SD, or as median and interquartile range (IQR), and differences in the distribution across groups were tested by one-way and two-way analysis of covariance tests adjusting for age and sex. Categorical variables were reported as percentage and differences in the distribution were tested by χ^2^ test. For correlations, the Pearson's test was used with Bonferroni correction. Determinants of masked hypertension were tested in multivariable logistic regression analyses using the BP response to standing either as a categorical or a continuous variable. The adjusted odds ratios (ORs) were provided with 95% confidence intervals (95% CI) and *P* values. A two-tailed probability value less than 0.05 was considered significant. To assess the predictive role of the BP reaction to standing, patients were also divided into four groups according to SBP reactivity (>6.5 or ≤6.5 mmHg) and 24-h urinary epinephrine/creatinine median level (>10.5 or ≤10.5 μg/g). To compare the four subgroups, the formula for Bonferroni correction was applied as follows: α_bonferroni_ = α_original_ / 6, where α_original_ is 0.05 and 6 is the total number of comparisons being performed among the four subgroups, giving a α_bonferroni_ = 0.05 / 6 = 0.0083. Thus, for the four-group comparisons, we rejected the null hypothesis of each individual test if the *P* value of the test was less than 0.0083.

Analyses were performed using Systat version 12 (SPSS Inc., Evanston, Illinois, USA) and MedCalc version 15.8 (MedCalc Software, Ostend, Belgium).

## RESULTS

At the baseline, lying office BP was at least 140/90 mmHg in all of the 1078 participants. Mean ± SD BP at entry was 145.8 ± 10.6/93.7 ± 5.9 mmHg and mean age was 33.2 ± 8.6 years. Due to the natural selection of people with high BP in this particular age range, there was a higher prevalence of men (*n* = 779, 72.3%). The mean orthostatic-supine BP difference (mean of six readings) was −2.6 ± 7.4 mmHg for SBP and 4.5 ± 5.4 mmHg for DBP. The distribution of orthostatic BP changes is shown in supplementary figures S1 and S2. Both SBP and DBP distributions had a positive skewness (*P* < 0.001).

### Follow-up blood pressure changes

After 3 months of follow-up, mean office BP fell to 140.4 ± 12.1/90.4 ± 8.5 mmHg (*P* < 0.001/ < 0.001). Average 24-h BP showed only a small decline from 131.0 ± 10.9/81.5 ± 8.2 to 130.6 ± 11.1/81.0 ± 8.4 mmHg (*P* = 0.079/*P* = 0.012). On the basis of follow-up office and average 24-h BPs, we could identify 120 individuals with both normal office and 24-h BPs, 166 individuals with white-coat hypertension, 168 individuals with masked hypertension and 624 individuals with sustained hypertension. Among the individuals with masked hypertension, 52 (31.3%) had normal office BP and 114 (68.7%) had high-normal BP. The characteristics of the study participants by BP category are reported in Table 1. Apart from office and ambulatory BP levels, only small differences in clinical characteristics were present between the four groups. However, the SBP decline from lying to standing was smaller in the masked hypertensive individuals than the other groups (*P* = 0.007 versus normotensive individuals, *P* = 0.037 versus white-coat hypertensive individuals, and *P* = 0.041 versus sustained hypertensive individuals). Another independent variable associated with the SBP response to standing was smoking (*P* = 0.014). The rate of hyperreactors to standing was 16.9% among the masked hypertensive individuals and was 4.2, 7.7 and 9.0%, respectively, in the normotensive, white-coat hypertensive and sustained hypertensive individuals (*P* = 0.002). The DBP response to standing did not differ between the masked hypertensive individuals and the other three groups. Both SBP and DBP responses to standing were better correlated with average daytime than night-time BP (table S1), even though the differences were small. The response of heart rate to standing was correlated with daytime but not with night-time SBP (table S1). In a sex- and age-adjusted ANCOVA, the orthostatic heart rate increase was greater in the hyperreactors than the normoreactors (mean ± SEM, 6.9 ± 0.5 versus 5.8 ± 0.2 bpm, *P* = 0.043). No between-group differences were found for both daytime and night-time heart rates (both *P* = n.s.).

**TABLE 1:** Baseline and follow-up characteristics of the participants grouped according to blood pressure status after 3 months of follow-up

Variable	Normal blood pressures (*N* = 120)	White-coat hypertension (*N* = 166)	Masked hypertension (*N* = 168)	Sustained hypertension (*N* = 624)	*P*
Age, years	31.8 ± 8.4	32.2 ± 8.1	32.8 ± 8.2	33.8 ± 8.7	0.030^a^
Sex, % men	64.2%	67.9%	70.5%	75.5%	0.029^a^
Baseline BMI (kg/m^2^)	24.4 ± 2.9	25.2 ± 4.0	24.8 ± 3.3	25.9 ± 3.4	0.001
Cigarette smokers	16.7%	15.5%	21.1%	23.2%	0.096
Alcohol drinkers	39.2%	41.7%	45.8%	49.1%	0.12
Baseline office SBP (mmHg)	143.1 ± 11.1	145.6 ± 10.4	141.4 ± 11.0	147.5 ± 9.9	<0.001
Baseline office DBP (mmHg)	91.3 ± 6.7	93.6 ± 5.3	91.8 ± 5.6	94.7 ± 5.5	<0.001
Baseline 24-h SBP (mmHg)	121.4 ± 9.8	125.2 ± 10.1	132.0 ± 8.8	134.2 ± 10.1	<0.001
Baseline 24-h DBP (mmHg)	75.1 ± 8.1	77.6 ± 7.0	81.3 ± 6.9	87.8 ± 10.0	<0.001
Baseline office heart rate (bpm)	75.1 ± 10.0	77.8 ± 9.4	72.9 ± 9.8	74.5 ± 9.6	<0.001
Baseline ortho SBP change (mmHg)	−3.8 ± 6.3	−3.1 ± 7.2	−0.9 ± 9.2	−2.6 ± 7.1	0.005
Baseline ortho DBP change (mmHg)	4.2 ± 5.8	3.6 ± 5.6	4.3 ± 4.9	4.9 ± 5.4	0.025
Baseline 24-h epinephrine/c (mg/g^b^)	10.5 (6.6–13.8)	10.6 (7.6–14.9)	11.1 (7.9–20.2)	10.3 (6.6–16.3)	0.046^c^
Baseline 24-h norepinephrine/c (mg/g^b^)	43.6 (29.5–68.0)	43.0 (32.4–62.9)	43.6 (29.5–68.0)	46.0 (31.1–68.8)	0.67^c^
FU office SBP (mmHg)	127.2 ± 7.5	143.0 ± 10.2	128.7 ± 7.3	145.3 ± 10.2	<0.001
FU office DBP (mmHg)	80.8 ± 6.7	92.3 ± 6.5	82.9 ± 5.9	93.8 ± 7.2	<0.001
FU 24h SBP (mmHg)	118.8 ± 7.0	120.5 ± 6.8	132.5 ± 8.8	135.1 ± 9.7	<0.001
FU 24h DBP (mmHg)	73.2 ± 5.7	74.2 ± 5.6	81.7 ± 7.9	84.2 ± 7.5	<0.001
FU ortho SBP change (mmHg)	−1.9 ± 8.0	−4.6 ± 9.2	1.8 ± 9.3	−3.3 ± 8.8	<0.001
FU ortho DBP change (mmHg)	6.7 ± 6.4	3.5 ± 7.0	6.8 ± 6.1	5.3 ± 7.0	<0.001

Data are mean values ± standard deviation or percentages. *P* values from ANCOVA, adjusted for age and sex. c, 24 h urinary creatinine; FU, after 3 months of follow-up; ortho, orthostatic.

a Unadjusted.

b Median (IQR).

c For log-transformed data adjusted for age, sex, alcohol and coffee use, smoking and physical activity habits.

**FIGURE 1:**
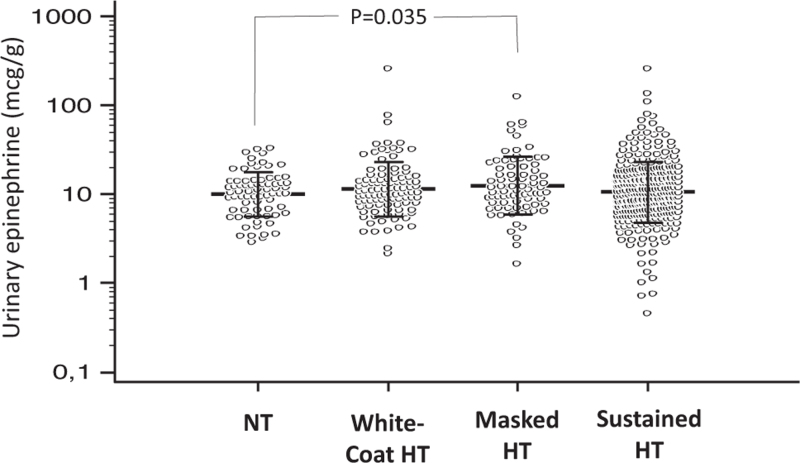
Baseline 24-h urinary epinephrine/creatinine in 591 participants stratified according to blood pressure status after 3 months of follow-up. Individual data points presented on a logarithmic scale are shown with mean ± SD. *P* value for masked hypertension versus rest of the population = 0.046 after logarithmic transformation of the data. *P* = n.s. for all other differences. HT, hypertensive individuals; NT, normotensive individuals;.

Urinary epinephrine/creatinine was higher in masked hypertensive individuals than the other three groups (Fig. 1). After adjustment for age, sex and lifestyle factors, the difference was significant versus the normotensive individuals (*P* = 0.035) and the rest of the population (*P* = 0.046), whereas no difference was found between normotensive and sustained hypertensive individuals (*P* = 0.64). No between-group differences were found for norepinephrine/creatinine.

After 3 months, mean postural BP changes (mean of three readings) were similar to those at baseline being −2.6 ± 9.1 mmHg for SBP and 5.4 ± 6.9 mmHg for DBP. The correlation coefficient between the orthostatic changes measured at the two time points was 0.29 (*P* < 0.001) for both SBP and DBP. After 3 months, an increase in SBP from lying to standing was found in the masked hypertensive individuals, whereas an SBP decline was observed in the other groups (Table 1) (*P* = 0.004 versus normotensive individuals, *P* < 0.001 versus white-coat hypertensive and sustained hypertensive individuals). The rate of hyperreactors to standing was 24.7% among the masked hypertensive individuals and was 8.3, 4.8 and 7.4%, respectively, in the other three groups (*P* < 0.001).

#### Association of orthostatic SBP reactivity with masked hypertension

**FIGURE 2:**
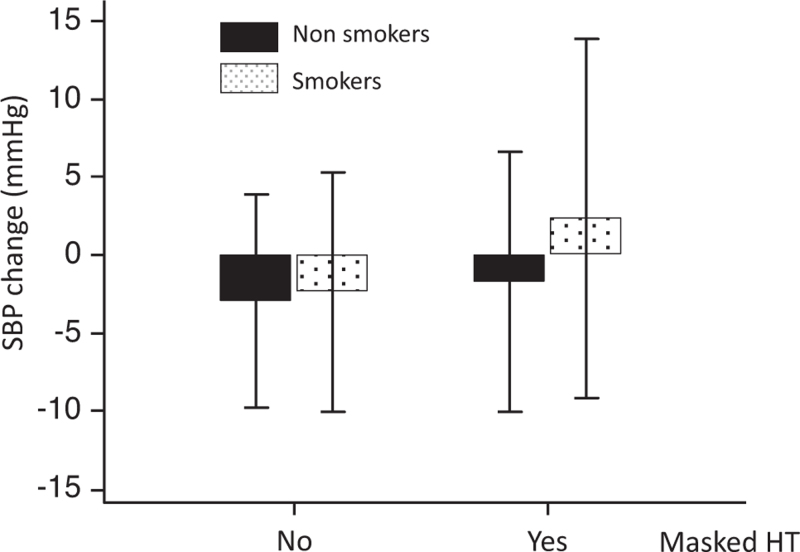
Age and sex-adjusted orthostatic SBP changes in 1078 participants stratified by masked hypertension (yes or no) and smoking (smokers versus nonsmokers). Masked hypertensives versus others, *P* < 0.001. Smokers versus nonsmokers, *P* = 0.002. Interaction of smoking with masked hypertension (*P* = 0.022). HT, hypertension.

**FIGURE 3:**
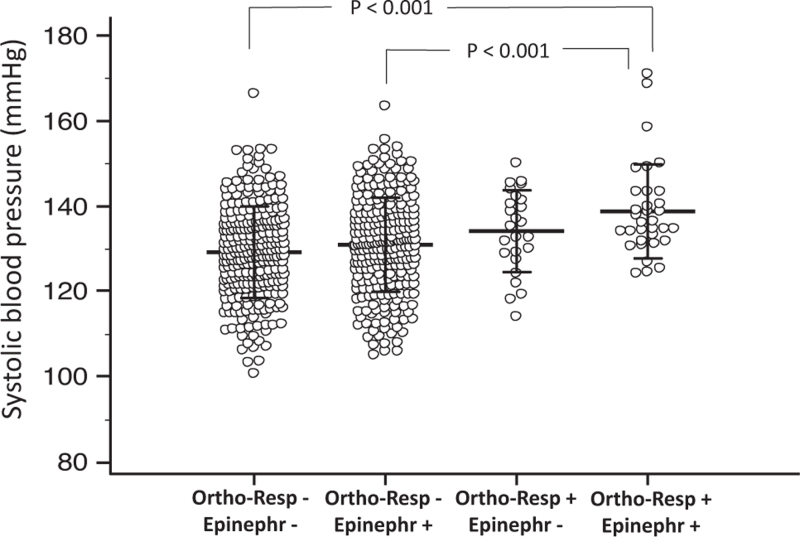
Average 24-h SBP in 591 participants stratified according to the systolic blood pressure response to standing and 24-h urinary epinephrine. Individual data points are shown with mean ± SD. *P* for ANCOVA <0.001. Epinephr -, 24-h urinary epinephrine/creatinine equal to or below the median; Epinephr +, 24-h urinary epinephrine/creatinine above the median; Ortho-Resp -, normal SBP response to standing; Ortho-Resp +, exaggerated SBP response to standing.

**FIGURE 4:**
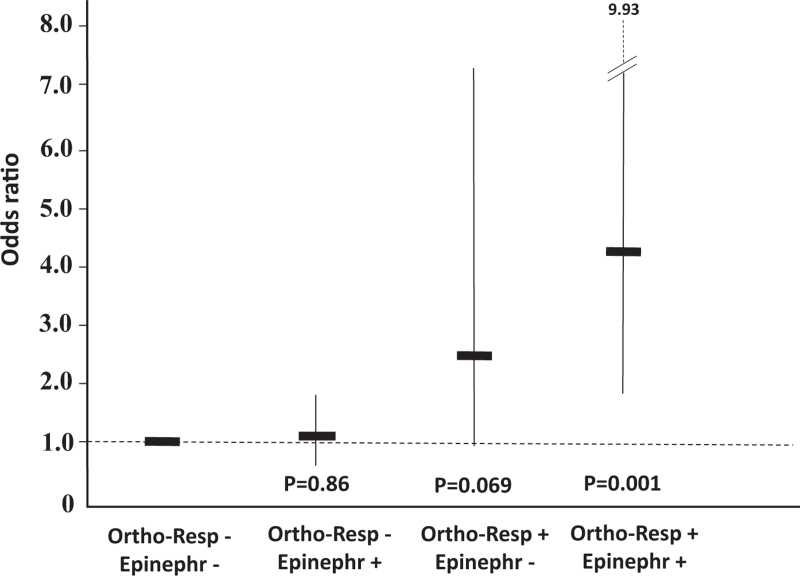
Odds ratios (95% confidence interval) for masked hypertension in 591 participants stratified according to the SBP response to standing and 24-h urinary epinephrine. Data are adjusted for age, sex, BMI and lifestyle factors. The Ortho-Resp -/ Epinephr - group was considered as the reference. Ortho-Resp - indicates normal SBP to standing; Ortho-Resp +, exaggerated SBP response to standing; Epinephr -, 24-h urinary epinephrine/creatinine equal to or below the median; Epinephr +, 24-h urinary epinephrine/creatinine above the median.

In a logistic regression analysis, including age, sex, BMI, smoking, alcohol and coffee use, and physical activity habits, the baseline SBP response to standing was associated with masked hypertension assessed after 3 months (Table S2). For the hyperreactors to standing, the OR was 2.45 (95% CI, 1.52–3.97, *P* < 0.001). In addition, in people with masked hypertension, the effect of smoking on SBP reactivity was amplified (*P* = 0.022 for interaction, Fig. 2). When the participants were grouped according to the reaction to standing and 24-h urinary epinephrine, 24-h SBP progressively increased from the group with normal reaction to standing and low epinephrine to the group with hyperreactivity and high epinephrine (Fig. 3). For the group of hyperreactors with high epinephrine, the OR was 4.21 (95% CI, 1.78–9.93) compared with the normoreactors with low epinephrine (Fig. 4). The between-group difference was significant (*P* = 0.001) also after the Bonferroni correction for multiple comparisons.

Similar results were obtained when masked hypertension was defined according to the combination of elevated daytime SBP and/or 24-h SBP. Hyperreactivity to standing had an adjusted OR of 2.45 (95% CI, 1.51–3.97, *P* < 0.001) for masked hypertension. The OR was lower when masked hypertension was defined as elevated night-time SBP and/or 24-h SBP combined (2.23; 95% CI, 1.43–3.47, *P* < 0.001) or as isolated masked asleep hypertension (OR, 1.88; 95% CI, 1.09–3.24, *P* = 0.024).

A relationship between the BP response to standing and masked hypertension was also found cross-sectionally when office BP and ambulatory BP were both measured after 3 months of follow-up. The association with masked hypertension was significant when the orthostatic BP change was considered either as a continuous (Table S2) or a binary categorical variable (OR, 4.78; 95% CI, 3.05–7.48, *P* < 0.001).

#### Group with orthostatic SBP decline

The decile of individuals at the lowest extreme of the distribution did not differ from the normoreactors or the hyperreactors with regard to age (*P* = 0.88) or sex (*P* = 0.56). The mean SBP decline in this subgroup was 15.2 ± 2.9 mmHg. The prevalence of masked hypertension was similar in the hyporeactors and the normoreactors (14.7 and 14.1%, respectively) and much lower than among the hyperreactors (27.2%, *P* = 0.002). In a fully adjusted logistic model, no association was found between hyporeaction to standing and masked hypertension (OR, 1.05; 95% CI, 0.59–1.86, *P* = 0.87).

The age and sex-adjusted level of epinephrine/creatinine in the hyporeactors, normoreactors and hyperreactors to standing are reported in supplementary figure S3. Epinephrine was higher in the hyperreactors than the normoreactors (*P* = 0.026), whereas no difference was found between the hyporeactors and normoreactors (*P* = 1.0). No between-group differences were found for norepinephrine/creatinine (*P* = 1.0).

## DISCUSSION

In this population of young to middle-age individuals screened for stage 1 hypertension, an exaggerated SBP response to standing was an independent determinant of masked hypertension in both the longitudinal and cross-sectional analyses, irrespective of whether the definition was based on average 24-h, daytime and/or night-time SBP. This association was stronger in the participants with higher 24-h epinephrine output. No association was found between hyporeactivity to standing and masked hypertension.

The prevalence of masked hypertension in the general population ranges from 8.5 to 16.6% and may be as high as 30.4% in populations with high-normal office BP [8,21,22]. Data from meta-analyses support the association of masked hypertension with target organ damage [23,24] and an increased risk of cardiovascular events and mortality, which is comparable to the risk of having sustained hypertension [25,26].

Unfortunately, a significant proportion of people with increased cardiovascular risk from masked hypertension remains undetected according to current diagnostic procedures. Several factors can selectively increase ambulatory BP, thereby increasing the likelihood of masked hypertension. Lifestyle factors, such as smoking, alcohol, physical inactivity, interpersonal conflicts, mental anxiety, job stress and high daytime physical activity [4,6,7], could thus be associated with masked hypertension. In a prospective study, risk factors for masked hypertension turned out to be male sex, age more than 40 years, BMI more than 27 kg/m^2^, smoking and alcohol intake more than six drinks/week [27]. EBPR to exercise has also been found to be associated with masked hypertension [9,28]. Among 75 individuals with EBPR, people with masked hypertension had increased BP change from baseline (61 versus 48 mmHg, *P* < 0.05). In a study of 61 normotensive individuals with EBPR, DBP measured at peak exercise was an independent predictor of masked hypertension [28].

However, stress testing is time-consuming, costly and not readily available at all physicians’ offices. Thus, a simpler test would be very helpful as a first-line screening tool. Previous research indicates that the BP response to standing was associated with masked hypertension [10,11]. A study of 304 treated hypertensive patients demonstrated that orthostatic hypertension (defined as a SBP increase ≥ 5 mmHg on standing) is an independent predictor of masked hypertension diagnosed with home BP measurement with an OR 3.65, 95% CI 1.27–10.51 [10]. In a cross-sectional study in a general population of 884 individuals assessed with ABPM, the frequency of masked hypertension was significantly greater in individuals who showed a postural SBP increase more than 10 mmHg 3 min after standing (52.1%) compared with controls (27.5%) with an OR of 3.01 (*P* = 0.001), irrespective of antihypertensive medication status. Our results obtained in a larger sample of untreated subjects screened for stage 1 hypertension, confirm those previous findings showing that the subjects with hyperreactivity to standing had a greater chance of having masked hypertension both in the longitudinal study and the cross-sectional analysis compared with the participants with normal orthostatic reaction. As the reproducibility of masked hypertension has been reported to be moderate also when using ABPM for its definition [29], we also examined the use of daytime and asleep BP and the combined use of BP status during the 24-h and during the ambulatory subperiods to categorize masked hypertension obtaining consistent results.

### Pathogenetic mechanisms

Orthostatic hypertension has been found to be associated with adverse cardiovascular outcomes both in young and elderly individuals and might be involved to some degree in the epidemiological relationship between masked hypertension and cardiovascular disease. One possible mechanism underlying the link between orthostatic hypertension and masked hypertension is sympathetic nervous system activity, which has been found to be elevated in both conditions [12,13]. In the present study, individuals with masked hypertension showed a higher level of urinary epinephrine measured over the 24 h compared with the normotensive individuals and the other hypertensive groups, suggesting increased adrenal medullary responsiveness to stressful stimuli. Our results are in keeping with those by Siddiqui *et al.*[30] who measured 24-h urine catecholamines in 156 treated hypertensive patients. Also in that study, sympathetic nervous system activation outside the clinic was higher in patients who had masked uncontrolled hypertension compared with those who had controlled BP both inside and outside the clinic. In agreement with previous data from the HARVEST [20], hyperreactors to standing had an increased level of urinary epinephrine confirming that adreno-medullary activation can also be present in people hyperreactive to standing and this might be the linchpin between these two clinical entities. That epinephrine facilitates, prejunctionally, norepinephrine release from the sympathetic nerve terminal has been actually shown by Floras *et al.*[31] who demonstrated in either normotensive or borderline hypertensive [32] individuals that epinephrine facilitates norepinephrine discharge and augments neurogenic vasoconstriction 30 min after it is infused. Several authors have suggested that endogenous epinephrine can induce norepinephrine release in human beings by this mechanism during and after episodes of sympatho-adrenal stimulation [33,34]. These studies thus support the concept that prejunctional beta receptor stimulation by epinephrine may facilitate noradrenergic transmission during orthostatic stress. Whether the higher 24-h epinephrine level found in our participants with masked hypertension reflects the higher epinephrine output found in our orthostatic hyperreactors or may also depend on hyperreactivity to other stressful situations of daily life is a matter for future research.

In addition, a hyperactive vascular α 1-adrenergic receptor responsiveness has been described in people with masked hypertension by Yano *et al.*[35] who documented an increased vascular reactivity to phenylephrine in 161 young to middle-age adults with this condition. This mechanism may also contribute to the greater orthostatic BP reaction found in people with masked hypertension. Alpha-adrenergic blockade was able to reduce the orthostatic BP increase in patients with orthostatic hypertension, suggesting that α-adrenergic hyper-reactive vascular disease is the underlying pathogenic condition [36,37].

Finally, orthostatic hyperreactivity may directly influence daytime BP, which mostly reflects BP measured in the standing or sitting positions, as suggested by the correlations of both BP and heart rate responses to standing with average daytime BP.

The current study extends previous research showing that there is an interplay between orthostatic hyperreactivity, enhanced sympatho-adrenergic activity and masked hypertension. An important role in this context may also be played by environmental factors. Smoking, a well recognized determinant of masked hypertension [7,27,38], showed a greater effect on the orthostatic BP changes in people with masked hypertension compared with the rest of the population. The above findings indicate that masked hypertension cannot be only considered as a precursor of sustained hypertension but a condition with a distinct pathogenetic background. Although the BP response to standing was correlated with ambulatory BP level, hyperreactivity to standing was more frequent in masked hypertension than sustained hypertension despite the higher ambulatory BP in the latter. In addition, epinephrine level was increased only in the masked hypertensive but not sustained hypertensive participants.

People with orthostatic hypotension had similar characteristics to those of the normoreactors to standing including the level of epinephrine and did not show any association with masked hypertension. Thus, in this population of young to middle-age individuals, a pronounced SBP decline after standing may represent the lower extreme of the postural change distribution rather than a distinct clinical entity.

#### Methodological issues

The majority of data showing an association between BP response to standing and risk of cardiovascular disease, including previous results from the HARVEST [20], were obtained from studies based on SBP [39,40]. Although diastolic orthostatic hypertension is a variant of orthostatic hypertension, only a few studies have investigated this condition [37]. In agreement with previous reports [10,11], in the present study, postural DBP changes were not associated with masked hypertension. Another methodological problem is that the procedures for evaluating orthostatic BP changes have been inconsistent in the literature, and thus, the method for measuring orthostatic BP changes has not been standardized [37,39]. Also, a widely accepted definition of hyperreactivity to standing has not been agreed upon and has ranged from a 5 to 20 mmHg increase in SBP [37,40,41]. In the present study, we used the 6.5 mmHg level obtained from the average of three SBP measurements within 3 min after standing up to define orthostatic BP hyperreactivity, in keeping with our previous report in a larger sample (upper decile of the orthostatic SBP change) [20]. This cut point is lower than that adopted in some studies, especially in older people [41], but is similar to that used in the Cardia study (5 mmHg) in individuals with a similar age to that of the HARVEST participants [40]. At any rate, the association between the postural BP change and masked hypertension was found with both the continuous and the categorical approaches.

#### Limitations

Several limitations of this study should be acknowledged. First, our participants with masked hypertension were not selected from a general population but from a population of individuals who were referred for stage 1 hypertension and whose office BP normalized within 3 months. Thus, the present results might not be generalizable to all people with masked hypertension. Second, another limitation of this posthoc analysis is the current lack of replication in an independent data set, which increases the risk of a false-positive finding. However, it should be noted that assessments of orthostatic BP changes [42] and of masked hypertension [43] were prespecified variables of interest in the HARVEST. Third, another possible limitation is that the presence of sleep apnoea was not assessed as a confounding factor in this investigation. However, sleep apnoea is rather uncommon in young low-risk individuals, with a higher prevalence among older adults and those with associated comorbid conditions. Fourth, we report data only from Whites, which may not be applicable to other ethnic groups. Finally, a further limitation may be the misclassification of lifestyle factors because their evaluation at baseline might not reflect health behaviours after 3 months.

In conclusion, our results indicate that among individuals screened for stage 1 hypertension, hyperreactivity to standing is an important determinant of masked hypertension. The OR was even quadrupled in people with a SBP response to standing more than 6.5 mmHg and high urinary epinephrine suggesting a role of sympathoadrenergic activity in the pathogenesis of this condition. An exaggerated SBP response to standing, especially in people with high-normal office BP, should thus be considered by the clinician as a key risk factor for masked hypertension. If confirmed with home BP monitoring or ABPM, the first therapeutic approach to masked hypertension associated with orthostatic hyperreactivity should be the improvement of lifestyle behaviours especially promoting avoidance of smoking and alcohol, which are implicated in the pathogenesis of both conditions. Although there have been no longitudinal clinical trials to evaluate the impact of antihypertensive drug treatment on cardiovascular events and mortality in patients with masked hypertension, consistent evidence showing increased cardiovascular risk in these patients favours the use of treatment despite lack of evidence. Whether specific antihypertensive treatments addressed to reduce BP reactivity to standing should be sought is a matter for future research.

## ACKNOWLEDGEMENTS

This study was funded by the Associazione ‘18 Maggio 1370’, San Daniele del Friuli, Italy.

### Conflicts of interest

None.

## Supplementary Material

Supplemental Digital Content
