# Supplementary material for: Blood pressure response to standing is a strong determinant of masked hypertension in young to middle-age individuals
Source: J Hypertens. 2022 Aug 23;40(10):1927–34. doi: 10.1097/HJH.0000000000003188 (PMC10860891; doi:10.1097/HJH.0000000000003188)
Supplement: Supplemental Digital Content [file jhype-40-1927-s001.docx]

**BLOOD PRESSURE RESPONSE TO STANDING IS A STRONG DETERMINANT OF MASKED HYPERTENSION IN YOUNG TO MIDDLE AGE SUBJECTS**

By P. Palatini et al.

**Table S1.** Correlations between the SBP, DBP, and heart rate reactions to standing and average daytime and night-time blood pressures at baseline.

| **Variable** | **Daytime**  **Coefficient** | **p-value*** | **Night-time**  **Coefficient** | **p-value*** |
| --- | --- | --- | --- | --- |
| SBP reaction | 0.104 | 0.002 | 0.096 | 0.005 |
| DBP reaction  Heart rate reaction | 0.151  0.079 | <0.001  0.028 | 0.112  0.049 | 0.001  0.332 |

SBP indicates systolic blood pressure; DBP, diastolic blood pressure. *With Bonferroni adjustment.

**Table S2.** Association of the systolic blood pressure reaction to standing with masked hypertension as binary variable in the longitudinal* and cross-sectional† analyses. Results from logistic regression analyses including baseline reaction and 3-month reaction one at a time.

| **Variable** | **Coefficient** | **S.E.** | **Wald χ^2^** | **p-value** |
| --- | --- | --- | --- | --- |
| Baseline SBP reaction* | 0.037 | 0.011 | 10.38 | 0.001 |
| 3-month SBP reaction† | 0.067 | 0.010 | 42.82 | <0.001 |

Data are adjusted for age, sex, body mass index, physical activity, smoking habits, coffee use and alcohol consumption. SBP indicates systolic blood pressure; S.E., standard error.

*Office SBP measured 3 months before the ambulatory blood pressure assessment

† Office SBP measured at the same time as the ambulatory blood pressure assessment

 N

Blood pressure change (mmHg) ,(mmHg)

**Figure S1.** Frequency histogram of the standing-lying systolic blood pressure difference in 1078 HARVEST participants. The distribution is skewed to the right with a coefficient of skewness of 0.26 (p<0.001).

Blood pressure change (mmHg) (mmHg)(mmHg)

**Figure S2.** Frequency histogram of the standing-lying diastolic blood pressure difference in 1078 HARVEST participants. The distribution is skewed to the right with a coefficient of skewness of 0.54 (p<0.001).

**Figure S3.** Baseline 24-hour urinary epinephrine/creatinine in 591 participants stratified according to their systolic blood pressure response to standing. Individual data points presented on a logarithmic scale are shown with mean ± SD. P-value for hyporeactors versus normoreactors = 1.00
